# The protective effects of sesamol and/or the probiotic, *Lactobacillus rhamnosus*, against aluminum chloride-induced neurotoxicity and hepatotoxicity in rats: Modulation of Wnt/β-catenin/GSK-3β, JAK-2/STAT-3, PPAR-γ, inflammatory, and apoptotic pathways

**DOI:** 10.3389/fphar.2023.1208252

**Published:** 2023-08-02

**Authors:** Karema Abu-Elfotuh, Heba Mohammed Refat M. Selim, Omnia Karem M. Riad, Ahmed M. E. Hamdan, Soha Osama Hassanin, Asmaa F. Sharif, Nouran Magdy Moustafa, Ayah M.H. Gowifel, Marwa Y. A. Mohamed, Ahmed M. Atwa, Sameh S. Zaghlool, Mahmoud Nour El-Din

**Affiliations:** ^1^ Clinical Pharmacy Department, Faculty of Pharmacy (Girls), Al-Azhar University, Cairo, Egypt; ^2^ Pharmaceutical Sciences Department, Faculty of Pharmacy, AlMaarefa University, Riyadh, Saudi Arabia; ^3^ Microbiology and Immunology Department, Faculty of Pharmacy, Al-Azhar University, Cairo, Egypt; ^4^ Pharmacy Practice Department, Faculty of Pharmacy, University of Tabuk, Tabuk, Saudi Arabia; ^5^ Biochemistry Department, Faculty of Pharmacy, Modern University for Technology and Information (MTI), Cairo, Egypt; ^6^ Forensic Medicine and Clinical Toxicology Department, Faculty of Medicine, Tanta University, Tanta, Egypt; ^7^ Clinical Medical Sciences Department, College of Medicine, Dar Al Uloom University, Riyadh, Saudi Arabia; ^8^ Basic Medical Science Department, College of Medicine, Dar Al Uloom University, Riyadh, Saudi Arabia; ^9^ Medical Microbiology and Immunology Department, Faculty of Medicine, Ain Shams University, Cairo, Egypt; ^10^ Pharmacology and Toxicology Department, Faculty of Pharmacy, Modern University for Technology and Information (MTI), Cairo, Egypt; ^11^ Biology Department, Faculty of Science, Imam Mohammad Ibn Saud Islamic University (IMSIU), Riyadh, Saudi Arabia; ^12^ Pharmacology and Toxicology Department, Faculty of Pharmacy, Egyptian Russian University, Cairo, Egypt; ^13^ Department of Pharmacology and Toxicology, Faculty of Pharmacy, University of Sadat City (USC), Menoufia, Egypt

**Keywords:** AlCl_3_, JAK-2/STAT-3, *Lactobacillus rhamnosus*, neurotoxicity, PPAR-γ, probiotic, sesamol, Wnt/β-catenin/GSK-3β

## Abstract

**Introduction:** Aluminium (Al) is accumulated in the brain causing neurotoxicity and neurodegenerative disease like Alzheimer's disease (AD), multiple sclerosis, autism and epilepsy. Hence, attenuation of Al-induced neurotoxicity has become a “hot topic“ in looking for an intervention that slow down the progression of neurodegenerative diseases.

**Objective:** Our study aims to introduce a new strategy for hampering aluminum chloride (AlCl3)-induced neurotoxicity using a combination of sesamol with the probiotic bacteria; *Lactobacillus rhamnosus (L. rhamnosus)* and also to test their possible ameliorative effects on AlCl_3_-induced hepatotoxicity.

**Methods:** Sprague-Dawley male rats were randomly divided into five groups (n = 10/group) which are control, AlCl_3_, AlCl_3_ + Sesamol, AlCl_3_ + *L. rhamnosus* and AlCl_3_ + Sesamol + *L. rhamnosus*. We surveilled the behavioral, biochemical, and histopathological alterations centrally in the brain and peripherally in liver.

**Results:** This work revealed that the combined therapy of sesamol and *L. rhamnosus* produced marked reduction in brain amyloid-β, p-tau, GSK-3β, inflammatory and apoptotic biomarkers, along with marked elevation in brain free β-catenin and Wnt3a, compared to AlCl_3_-intoxicated rats. Also, the combined therapy exerted pronounced reduction in hepatic expressions of JAK-2/STAT-3, inflammatory (TNF-α, IL-6, NF-κB), fibrotic (MMP-2, TIMP-1, α-SMA) and apoptotic markers, (caspase-3), together with marked elevation in hepatic PPAR-γ expression, compared to AlCl_3_ -intoxicated rats. Behavioral and histopathological assessments substantiated the efficiency of this combined regimen in halting the effect of neurotoxicity.

**Discussion:** Probiotics can be used as an add-on therapy with sesamol ameliorate AlCl_3_ -mediated neurotoxicity and hepatotoxicity.

## Graphical Abstract

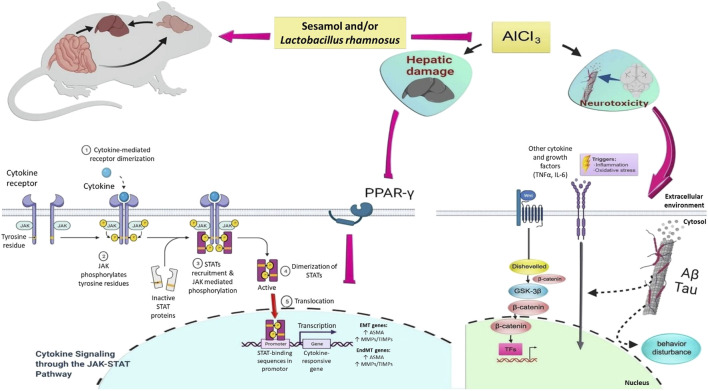

## 1 Introduction

Aluminum (Al), an environmental toxicant, is considered a potential etiological factor causing neuropathological and neurochemical alterations, together with neurobehavioral abnormalities. Al-based products are used in many fields, including industry, medicine, and agriculture (Klotz et al., 2017). In addition, they are widely involved in food manufacturing and daily necessities, such as food additives, antiperspirants, Al cooking utensils, Al foil, water purifiers, and some vaccines, which largely increase the risk of exposure to Al in humans. Al can negatively impact human health, where it is distributed mainly in the brain, liver, and kidney tissues (Kandimalla et al., 2016; Xu et al., 2017; Alasfar and Isaifan, 2021)**.** Hence, exposure or intake of Al for a long time produces toxic effects in humans and animals and may result in conditions such as neurotoxicity, hepatotoxicity, kidney disease, and Alzheimer’s disease (AD) (Mailloux et al., 2011; Krause et al., 2020).

Researchers have recently found increased levels of Al in the brain tissue of people suffering from epilepsy, autism, or AD (Alasfar and Isaifan, 2021). Thus, Al toxicity may be responsible for neurotoxicity and neurodegenerative disorders, including AD, autism, or multiple sclerosis and their related manifestations, such as memory loss and dementia.

A growing body of evidence suggests that amyloid-beta (Aβ) deposition is not only a central player in the pathogenesis of AD, but it also has a pivotal role in the etiopathogenesis of other neurodegenerative disorders, including multiple sclerosis, autism, and epilepsy (Sokol et al., 2019; Romoli et al., 2021; Lopresti, 2022). Physiologically, there is a balance between the rate of production of Aβ and its rate of degradation (Baranello et al., 2015). The degradation of Aβ occurs mainly through the liver (Maarouf et al., 2018). Actually, the liver, a vital organ for the metabolism of chemicals, metabolizes more than 60% of Aβ, thus limiting Aβ accumulation. Given these facts, upon Al-intoxication, the liver, the major organ for Al accumulation, can be injured, resulting in impairment in liver metabolism that could lead to further Aβ accumulation in the brain and hence fostering the progression of neurodegenerative diseases. Furthermore, liver dysfunction may play a role in the progression of neurodegenerative diseases by acting as a source of pro-inflammatory mediators (Wiseman et al., 2015; Estrada et al., 2019).

It is interesting to note that recent research studies have revealed synergistic interactions between Aβ and the tau protein in several neurodegenerative disorders (Sengupta and Kayed, 2022). Al neurotoxicity and the accumulation of Aβ result in neuroinflammation, neurodegeneration, and neuronal apoptosis. In fact, Aβ further stimulates the expression and production of several pro-inflammatory mediators, such tumor necrosis factor-α (TNF-α), interleukin-1β (IL-1β), and interleukin-6β (IL-6β), by the activated microglia, which in turn trigger the hyperphosphorylation of tau protein in the brain that forms neurofibrillary tangles (NFTs), resulting in neuronal cell death (Ghosh et al., 2013; Meraz-Ríos et al., 2013; González-Domínguez et al., 2015; Calsolaro and Edison, 2016; Cao et al., 2016; Domingues et al., 2017). There is ample evidence that the dysregulation of the Wnt3a/β-catenin/GSK-3β signaling pathway has a crucial role in both the onset and progression of neurodegenerative disorders resulting from the neurotoxic effects of Al (Li et al., 2020), where glycogen synthase kinase-3β (GSK-3β) phosphorylates tau, contributes to the development of Aβ (Hernandez et al., 2013), and initiates neurodegeneration (Jia et al., 2019; Lauretti et al., 2020).

It has been documented that long-term Al exposure is associated with alteration in the gut microbiota, leading to gut dysbiosis that elevates systemic inflammation and disrupts the gut–brain axis, thus triggering neuroinflammation and neurotoxicity and enhancing the progression of neurodegenerative diseases via dysregulation in the gut–brain axis (Zhai et al., 2017).

Several research studies have looked into potential protective measures in light of Al’s significant propensity for neurotoxicity and its role in the emergence of neurodegenerative and neurodevelopmental diseases. As inflammation, oxidative stress, apoptosis, mitochondrial dysfunction, and endoplasmic reticulum stress all play a part in Al neurotoxicity, it is expected that the majority of protective drugs will exert their protective effects by acting as antioxidants and anti-inflammatory agents. The current protective agents include chelators to bind Al; polyphenols; or polyphenol-rich phytoextracts as antioxidants (Skalny et al., 2021).

To date, there is a growing interest in alternative medicine because herbs and herbal-derived products possess myriad actions, such as antioxidant, anti-inflammatory, anti-amyloid, and anti-apoptotic activities (Kumar and Arti Singh, 2017). Among the widely used, potentially editable nutritional and medicinal herbs in most countries are sesame seed. It has various medicinal uses, such as in the dry couch and asthma as a bronchodilator, ulcers as an anti-inflammatory agent, urinary tract diseases and wound healing as an antimicrobial agent, brain diseases such as migraine and vertigo as a neuroprotective agent, congestive heart failure as a cardioprotective agent, and liver diseases as a hepatoprotective agent (Mili et al., 2021). It contains sesame lignans (including sesamol), which have an inhibitory effect on cholesterol 24S-hydroxylase (CYP46A1), which is a brain-specific cytochrome P450 and plays a significant role in the cholesterol catabolism in the brain tissues, preventing cerebral ischemia in the middle cerebral arteries and inhibiting the cerebral apoptotic pathway (Du et al., 2022). Sesamol is a promising candidate to attenuate neurotoxicity and neuroinflammation due to its anti-inflammatory activity (Sachdeva et al., 2015; Castro-González et al., 2020)**.** Sesamol has neuroprotective properties and can help with cognitive deficits and anxiety. Interestingly, sesamol has been documented to prevent Aβ accumulation, change the microbiota of the stomach, and improve the microbial metabolite output (Yuan et al., 2019). Sesamol prevents Al-induced neurotoxicity through its anti-inflammatory, antioxidant, and anti-apoptotic effects (Abou-Zeid et al., 2021; Du et al., 2022).

The gut–brain axis has received a lot of attention for its effect on the healthy function of the central nervous system (Dempsey et al., 2019). Many research studies have shown that probiotic therapy can be used for different organophosphorus toxins, metal toxicity, and many medication toxicities (Feng et al., 2019). The proven molecular mechanisms of such a detoxification process, even in low-concentrations, of the toxins include that probiotics precipitate these compounds (Chen et al., 2022). Improving the gut microbiota with probiotics could be considered a promising therapy for neurodegenerative diseases, especially those triggered by neurotoxicity. Probiotic bacteria modulate the gut–brain axis. Owing to their anti-inflammatory and antioxidant activities, probiotics can help enhance memory functions, improve cognitive functions, and hence halt the development of neurodegenerative diseases, including AD (Arora et al., 2020). *Lactobacillus plantarum* is an example of probiotic bacteria that is found to restore gut microbiota in the AD model and protect from induced eye neurodegeneration in the AD model (Tan et al., 2020). It has been reported recently that *Lactobacillus casei* administration prevented neuronal death, decreased the accumulation of Aβ, and hyperphosphorylated p-tau. These probiotic bacteria protect against neuronal death through their antioxidant, anti-inflammatory, and immune-modulating properties and their ability to restore gut microbiota dysbiosis caused by Al neurotoxicity (Qiao et al., 2022)**.** Metal chelation, a classical therapy for metal toxicity such as copper and aluminum, could be achieved through probiotic bacteria, e.g., *L. rhamnosus* (*Lactobacillus rhamnosus)* (Gaisawat et al., 2019)**.**

Based on the aforementioned data, the current study aims to evaluate and provide mechanistic insights on the protective effects of sesamol and/or the probiotic bacteria *L. rhamnosus* against the behavioral, biochemical, and histopathological alterations centrally in the brain and peripherally in the liver of aluminum chloride (AlCl_3_)-intoxicated rats.

## 2 Materials and methods

### 2.1 Drugs and dosage

Aluminum chloride hexahydrate (AlCl_3_⋅6H_2_O; CAS number 7784-13-6) and sesamol (CAS number 533-31-3) were purchased from Sigma-Aldrich Chemical Co. (St. Louis, MO, United States).

### 2.2 Bacterial strain and culture conditions

A standard strain of *L. rhamnosus* (ATCC 7469) was used in the current study. It was cultured in de Man Rogosa Sharpe (MRS) agar (Oxoid, England) and broth at 37°C for 24 h under aerobic conditions (Zhang et al., 2015). A suspension of *L. rhamnosus* (1 × 10^6^ CFU) was prepared in sterile normal saline for feeding the rats.

### 2.3 Animals

Fifty male Sprague–Dawley rats weighing 300–320 g and aged 8 months were supplied from Nile Co., Cairo, Egypt. They were kept in stainless steel cages under typical housing conditions (25°C temperature and 12-h dark and light intervals). Rats were fed *ad libitum*. The Animal Care and Use committee of the Faculty of Pharmacy, Al-Azhar University, authorized all experimental protocols [ethical permission number (216/2021)]. This is consistent with the guidelines outlined in the “Guide for Care and Use of Laboratory Animals,” published by the National Institutes of Health (NIH Publications No. 8023, revised 1978).

### 2.4 Experimental design

**FIGURE 1:**
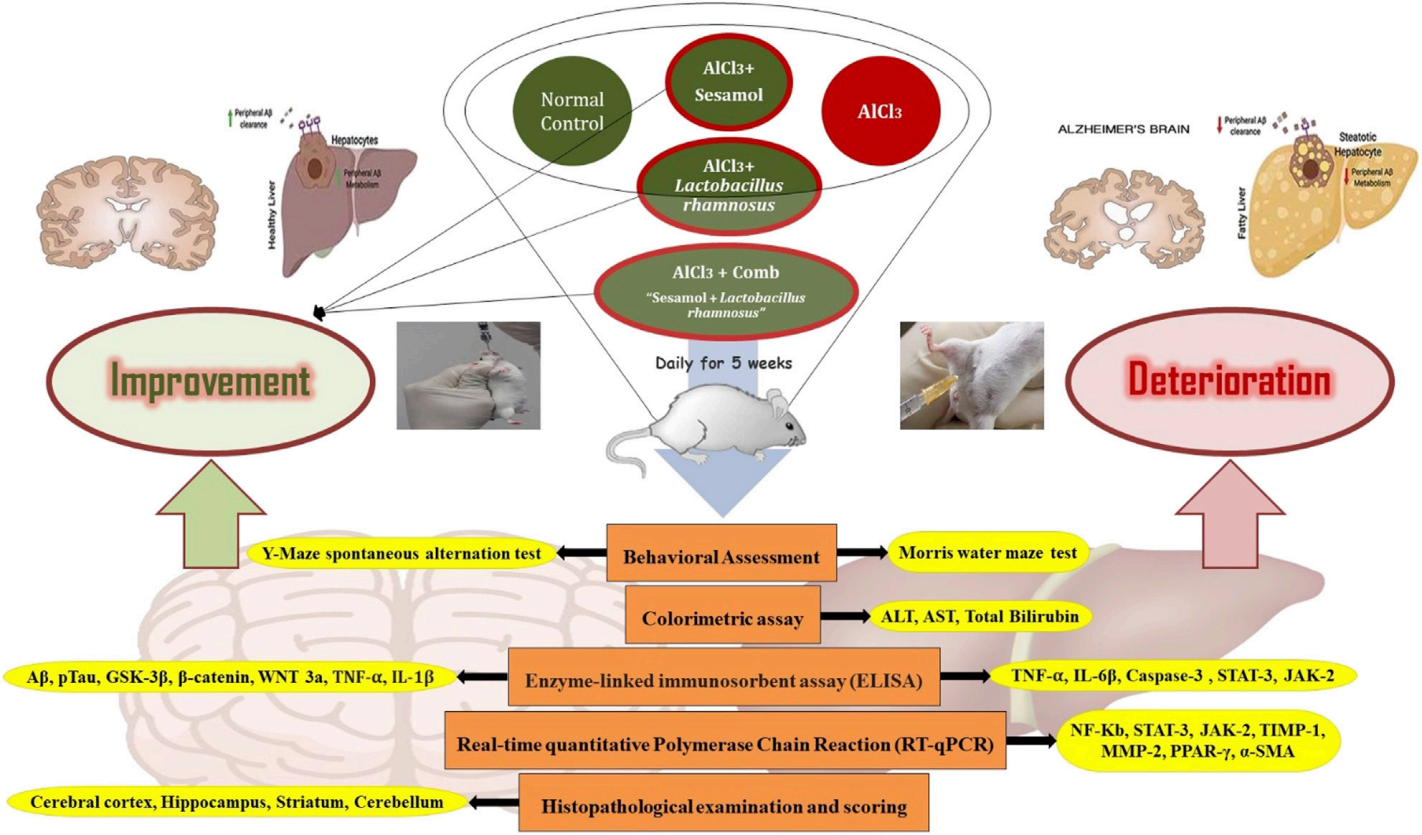
Schematic diagram for the experimental design and the tests conducted for each group.

Rats were randomly divided into five groups (*n* = 10). Group (I) received both 1 mL of distilled water orally (*p.o.*) and 1 mL of saline injection intraperitoneally (*i.p*) daily for 5 weeks and was considered the control group. Group (II) received daily AlCl_3_ (70 mg/kg/day *i.p.* for 5 weeks) (Ali et al., 2019). Group (III) received daily AlCl_3_ (70 mg/kg/day *i.p.* for 5 weeks) and daily sesamol (50 mg/kg/day *p.o.*; freshly dissolved in distilled water) (Singh et al., 2020) and was named the AlCl_3_ + sesamol group. Group (IV) received daily AlCl_3_ (70 mg/kg/day *i.p* for 5 weeks) and daily *L. rhamnosus* (1 × 10^6^ CFU/day *p.o.*) for 5 weeks (Patel et al., 2020) and was named the AlCl_3_ + *L. rhamnosus* group. Furthermore, group (V) received daily AlCl_3_ (70 mg/kg/day *i.p* for 5 weeks) and a combination of sesamol (50 mg/kg/day *p.o.*; freshly dissolved in distilled water) and *L. rhamnosus* (1 × 10^6^ CFU/day *p.o.*) for 5 weeks as the solo-treated groups and was named the AlCl_3_ + combination group. Behavioral tests were performed 24 h after the last dose (as depicted in Figure 1).

### 2.5 Behavioral assessment before and after treatment with sesamol and/or probiotic *L. rhamnosus*

Supplementary Figure S1 explains the behavioral tests and the indication for these tests.

#### 2.5.1 Morris water maze test

The Morris water maze (MWM) test was used to track learning memory and cognitive functions as previously described (Vorhees and Williams, 2006). Briefly, the MWM device is a round black pool with four equal quadrants (150 cm in diameter and 62.5 cm in height). The pool was filled to a depth of 40 cm with water that was kept at a constant temperature of 20°C ± 1°C. A round platform with a diameter of 13 cm was buried approximately 1 cm beneath the ink-stained water surface (Morris, 1984). The MWM test is a hippocampus-dependent spatial learning task where rats must learn how to identify visible or invisible platforms in a pool of water by utilizing visual cues around the maze. In the first phase (escape latency), each rat was permitted to swim freely for 1 minute for 4 days (the last 4 days of the experiment) in a row from a different quadrant till they found the concealed platform, staying for 20 s on the platform to get a good sense of the location. Afterward, they were pulled off. The escape latency (the time taken to discover the secret platform) was measured in seconds on the last day of the experiment. Rats swam for 60 s in the absence of the training platform in the second phase (memory probe phase), and the time taken in the target quadrant was measured in seconds (Morris, 1984).

#### 2.5.2 Y-maze spontaneous alternation test

As previously described (Hritcu et al., 2012), a black wood Y-maze with three arms (35 cm long, 25 cm high, and 10 cm broad) with asymmetrical triangular core regions, each labeled A, B, or C, was employed on the last day of the study to determine the spatial working memory that is reflected in spontaneous alternation behavior (Hritcu et al., 2012)**.** For 8 minutes, rats were put at the end of one arm and permitted to freely navigate through the maze. It was considered an entrance when the rat’s hind paws were fully within the arm. In each of the three arms, spontaneous alteration behavior was defined as a series of successive entries into an overlapping triplet combination pattern (CAB and ABC). Before the next animal was tested, the maze was washed with a 10% ethanol solution and dried with a towel to eliminate any lingering scents. According to the number of alternations and total arm entries, we calculated the spontaneous alternation percentage (SAP) using the following equation:

SAP % = [number of alternations/ (total number of arm entries)] × 100 (Hritcu et al., 2012)**.**

### 2.6 Tissue sampling and preparation

As previously described (Goss, 2009), after 24 h of the last behavioral test, fasted rats were anesthetized with ketamine (80 mg/kg *i.p.*) and euthanized by cervical translocation. The brain and liver of each rat in each group were rapidly separated and cleaned with ice-cold isotonic saline. Four brains and livers from each group were fixed overnight in 10% neutral buffered formalin for histopathological investigations. Each of the remaining six brains and livers per group was longitudinally cut into two equal parts; one part was homogenized in 1 M phosphate buffer (pH 7.4) and centrifuged at 1,800 *g* for 10 min at 4°C, where the supernatant was then used for various biochemical measurements, like enzyme-linked immunosorbent assay (ELISA) assessments. The other part was kept at −80°C, in which a solution was preserved (PBS-inhibitor proteases-DNAses) to be used in real-time PCR analyses.

### 2.7 Biochemical analysis before and after treatment with sesamol and/or probiotic *L. rhamnosus*

#### 2.7.1 Real-time quantitative polymerase chain reaction (RT-qPCR)

Assessment of mRNA levels of brain Bcl-2/Bax and the housekeeping gene (β-actin) in brain tissue was performed using qPCR using Applied Biosystems step one plus equipment (Glare et al., 2002; Briton-Jones et al., 2006). Measurement of mRNA levels of peroxisome proliferator-activated receptor gamma (PPAR-γ), nuclear factor kappa B (NF-κB), Janus kinase-2 (JAK-2), signal transducers and activators of transcription (STAT-3), matrix metalloproteinase-2 (MMP-2), tissue inhibitor matrix metalloproteinase-1 (TIMP-1), alpha-smooth muscle actin (α-SMA), and the housekeeping gene (β-actin) in liver tissue was carried out by RT-qPCR using Applied Biosystems step one plus equipment. Real-time PCR was carried out as outlined previously (Glare et al., 2002; Sayed-Ahmed et al., 2010). Total RNA from tissues was extracted using TRIzol (Life Technologies) according to the manufacturer’s instructions and purified using the RNeasy Mini unit (Qiagen, Hilden, Germany). Afterward, the reverse translation was performed using the Superscript III pack (Life Technologies). The complementary DNAs were broken down by quantitative continuous PCR. Additionally, using the SYBR Green PCR Supermix unit, relative messenger RNA (mRNA) expressions were quantified and studied using constant polymerase chain reaction (RT-PCR) (Bio-Rad Laboratories, Hercules, CA, United States). Primer sequences are shown in Table 1.

**TABLE 1:** Forward and reverse sequences of the primers used in the RT-PCR.

Gene	Primer pair sequence	Gen ID	Number of base pairs
*PPAR-γ*	F: 5′-AAG​GCT​GCA​GCG​CTA​AAT​TC-3′	NM_001145366	625 bp
	R: 5′-GCA​AGG​CAC​TTC​TGA​AAC​CG-3′		
*NF-κB*	F: 5′-CAT​TGA​GGT​GTA​TTT​CAC​GG-3′	NM_199267	284 bp
	R: 5′-GGC​AAG​TGG​CCA​TTG​TGT​TC- 3′		
*JAK-2*	F: 5′-TCC​ACC​CAA​TCA​TGT​CTT​CCA-3′	NM_031514	856 bp
	R: 5′-ATG​GTG​TGC​ATC​CGC​AGT​TA-3′		
*STAT-3*	F: 5′-GCT​GAG​GTA​CAA​TCC​CGC​TC-3′	NM_012747	994 bp
	R: 5′-TTG​TTG​GCG​GGT​CTG​AAG​TT-3′		
*MMP-2*	F: 5′-GTG​CCC​AAA​GAA​AGG​TGC​TG-3′	NM_031054	518 bp
	R: 5′-CAG​GAA​GCC​GAT​GAC​TTG​GT-3′		
*TIMP-1*	F: 5′-AGA​CAC​GCT​AGA​GAT​ACC​ACG-3′	NM_053819	142 bp
	R: 5′-CCA​GGT​CCG​AGT​TGC​AGA​AA-3′		
*α-SMA*	F: 5′-ACT​ACT​GCC​GAG​CGT​GAG​AT-3′	NM_031004	402 bp
	R: 5′-AAG​GTA​GAC​AGC​GAA​GCC​AA-3′		
*Bax*	F: 5′-ATG​TTT​TCT​GAC​GGC​AAC​TTC-3′	NM_017059	566 bp
	R: 5′-AGT​CCA​ATG​TCC​AGC​CCA​T-3′		
*Bcl2*	F: 5′- CTA​CGA​GTG​GGA​TGC​TGG​AG-3′	NM_016993	105 bp
	R: 5′- TTC​TTC​ACG​ATG​GTG​AGC​G-3′		
*β-Actin*	F: 5′-CCG​TAA​AGA​CCT​CTA​TGC​CA- 3′	NM_031144	299 bp
	R: 5′-AAG​AAA​GGG​TGT​AAA​ACG​CA- 3′		

The reaction was performed in triplicate for each sample, with at least three independent runs. Real-Time StatMiner was used to assess the expression levels (Integromics, Madrid, Spain). Fold changes were calculated using relative quantification using β-actin levels as an internal control.

#### 2.7.2 Enzyme-linked immunosorbent assay (ELISA)

The brain contents of amyloid-β (Aβ) (catalog no. MBS702915, MyBioSource, San Diego, United States), phosphorylated tau (p-tau) (catalog no. MBS725098, MyBioSource, San Diego, United States), GSK-3β (catalog no. MBS3808332, MyBioSource, San Diego, United States), JAK-2 (catalog no. MBS2019409, MyBioSource, San Diego, United States), STAT-3 (catalog no. MBS760293, MyBioSource, San Diego, United States), β-catenin (catalog no. K3383, Biovision Inc.), Rat Wnt3a ELISA kit (catalog no. orb555678, Biorbyt Ltd., United Kingdom), TNF-α (catalog no. RTA00, Quantikine, R&D systems Europe, Abingdon, United Kingdom), IL-6β (catalog no. BMS625TEN, ThermoFisher Scientific Inc.), IL-1β (catalog no. BMS630, ThermoFisher Scientific Inc.), and caspase-3 (catalog no. CASP3C, ThermoFisher Scientific Inc.) were measured in brain homogenate using ELISA kits according to the manufacturer’s instructions.

### 2.8 Histopathological examination and scoring before and after treatment with sesamol and/or probiotic *L. rhamnosus*

As previously described by Suvarna et al. (2019), briefly, the brain and liver of four representatives, randomly chosen rats from each group, were separated and fixed in 10% neutral buffered formalin for 24 h in a hot air oven at 56°C. Afterward, specimens were dried using a series of decreasing alcohol dilutions, cleaned in xylene, and embedded in paraffin blocks. The cerebral cortex, hippocampus subiculum, hippocampus fascia dentate, and striatum paraffin blocks were cut at 4 mm thickness using a hammer microtome. Tissue slices were collected on glass slides, deparaffinized in xylene, and hydrated in increasing percentages of ethyl alcohol before staining with the hematoxylin and eosin (H & E) stain. A grading system for brain and liver tissues was used to quantify the lesion, where - represents zero or a normal histological status without changes (0%), (+) refers to minimal changes or less than 25% of examined tissue sections have changes, (++) equal to moderate changes or about 25–45%, while (+++) refers to severe changes or more than 50% of examined tissue sections having changes (Gibson-Corley et al., 2013).

### 2.9 Serum sampling and preparation

Blood samples from each rat were taken through the eye puncture and put into serum separator tubes before scarification. The tubes were let to stand for 30 min, after which they were centrifuged for 15 min at 3,000 rpm. The serum was then collected and stored at −20°C until the biochemical parameters under investigation were determined (Abu-Elfotuh et al., 2021).

Hepatic function estimation: using colorimetric assay kits (Biomed-diagnostics, Cairo, Egypt), serum alanine aminotransferase (ALT), serum aspartate aminotransferase (AST), and total bilirubin were calculated (Malloy and Evelyn, 1937; Burtis and Ashwood, 1994).

### 2.10 Statistical analysis

An analysis of variance (ANOVA) was used to evaluate the data, followed by Tukey’s *post hoc* test. All results were reported as the mean ± SEM. A two-way ANOVA was used to assess the statistical analysis of the escape latency time for 4 days in the MWM test. For all statistical analyses, GraphPad Prism software (version 8.0, GraphPad Software Inc., San Diego, CA, United States) was used, and a *p* value less than 0.05 was considered statistically significant.

## 3 Results

In this work, the effects of sesamol, *L. rhamnosus*, and sesamol + *L. rhamnosus* were studied on the control groups, but none of these three groups showed significant differences from the control group in any of the measured parameters. Thus, these results were not displayed to avoid data complexity.

### 3.1 Effect of administration of sesamol and/or *L. rhamnosus* on the brain contents p-tau and Aβ in AlCl_3_-intoxicated rats

**FIGURE 2:**
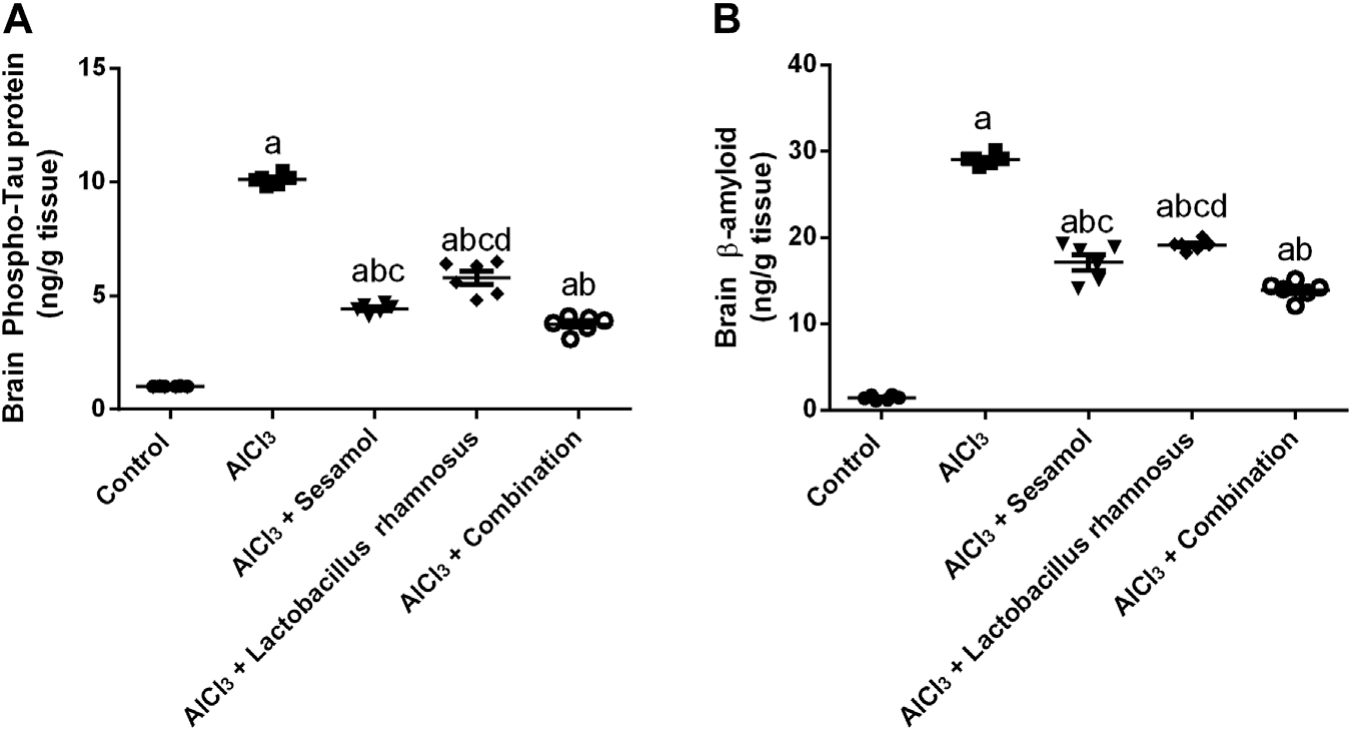
Effect of administration of sesamol and/or *Lactobacillus rhamnosus* on the brain contents of p-tau **(A)** and Aβ **(B)** in AlCl_3_-intoxicated rats. Values are shown as the mean (*n* = 6) ± SEM. One-way ANOVA was used for statistical analysis, followed by Tukey’s *post hoc* test. Comparison of (a) control, (b) AlCl_3_, (c) sesamol + *Lactobacillus rhamnosus*, and (d) *sesamol* treated groups; *p* < 0.05. Aβ, amyloid beta.

AlCl_3_-intoxicated rats showed an elevation in brain levels of both phosphorylated tau (p-tau) protein (Figure 2A) and amyloid-β (Aβ) (Figure 2B) by 10 and 30 times, respectively, in comparison with the normal control group. Administration of sesamol alone markedly reduced the levels of both p-tau protein and Aβ by 50% and 35%, respectively, compared with the AlCl_3_-intoxicated rats. Treatment with *L. rhamnosus* alone decreased levels of both p-tau protein and Aβ by 38% and 32%, respectively, compared with the AlCl_3_-intoxicated rats. Nevertheless, treatment with both sesamol and *L. rhamnosus* significantly reduced the levels of p-tau protein and Aβ by 61% and 51%, respectively, compared with the AlCl_3_-intoxicated rats.

### 3.2 Effect of administration of sesamol and/or *L. rhamnosus* on the behavioral alterations in AlCl_3_-intoxicated rats

**FIGURE 3:**
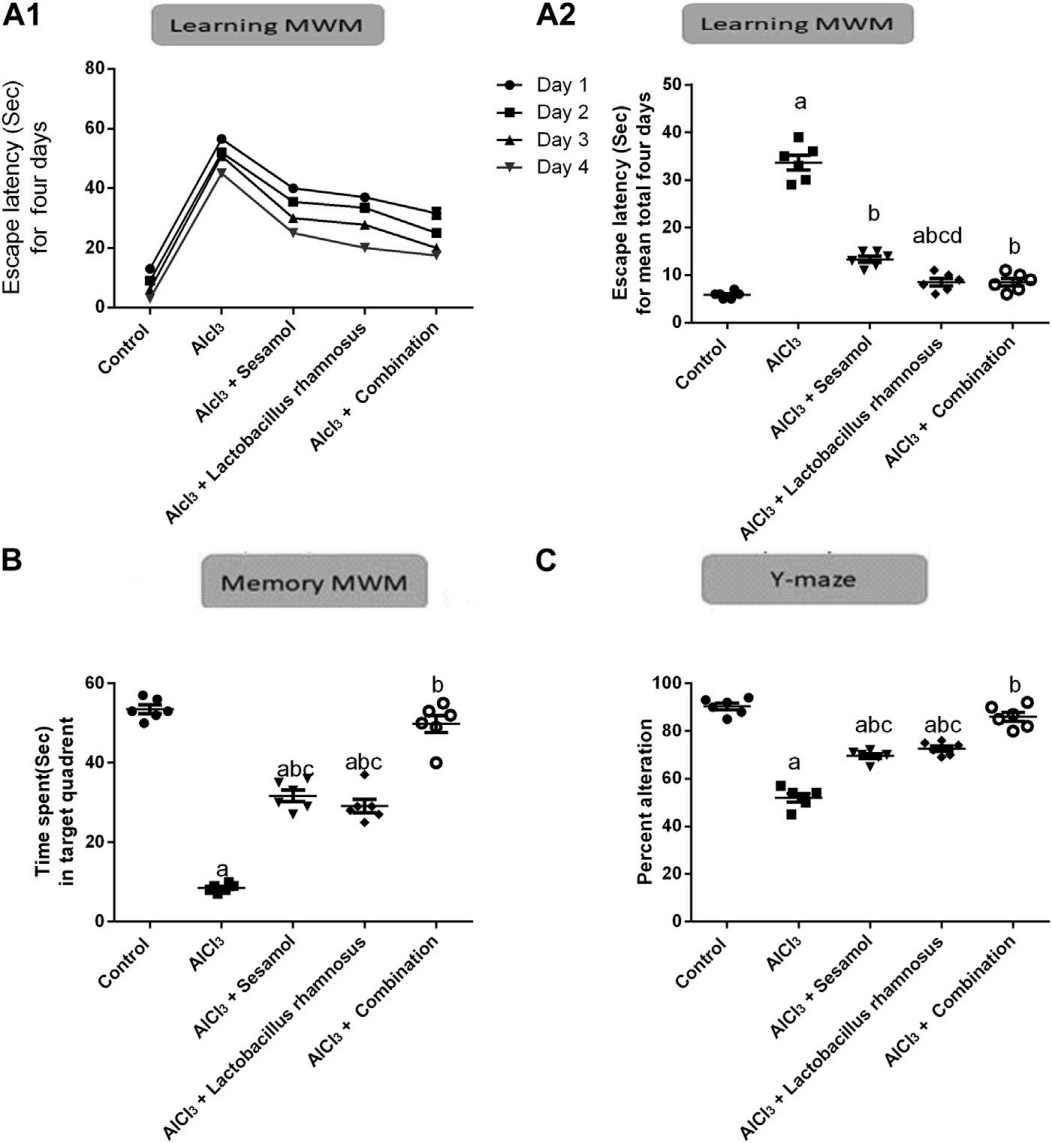
Effect of administration of sesamol and/or *Lactobacillus rhamnosus* on the behavioral alterations in AlCl_3_-intoxicated rats: learning Morris’s water maze (learning-MWM) **(A1,A2)**, memory Morris’s water maze (memory-MWM) **(B)**, and Y-maze spontaneous alternation tests **(C)**. Values are presented as the mean (*n* = 6) ± SEM. Statistical analysis was carried out using the two-way ANOVA of the escape latency time for 4 days in the MWM test **(A1)**.One-way ANOVA followed by Tukey’s *post hoc* test for **(A2,B,C)**. Comparison of (a) control, (b) AlCl_3_, (c) sesamol + *Lactobacillus rhamnosus*, and (d) sesamol treated groups; *p* < 0.05.

As depicted in Figure 3, AlCl_3_-intoxicated rats presented reduced spatial learning and memory in the MWM, as indicated by a significant increase in escape latency time for 4 days and for a total of 4 days (Figure 3A1, A2) (the learning task; time to reach the platform) and a decrease in time spent in the target quadrant (Figure 3B) (the memory task; 2 h after the last trial on day 4) as compared to control rats. AlCl_3_ caused an increase in the escaping latency time by 5.8 times on day 1 compared to the control group (Figure 3A). Moreover, treatment with AlCl_3_ reduced the memory task by nine times compared with the control group (Figure 3B). Likewise, AlCl_3_ caused reduced special memory, as shown in the Y-maze (Figure 3C), in AlCl_3_-intoxicated rats by 54% compared with the control group. On the contrary, treatment with sesamol enhanced both the learning and memory tasks, by reducing the escaping latency by 28% and increasing the time spent in the target quadrant about three times compared with AlCl_3_-intoxicated rats. Furthermore, treatment with sesamol enhanced the spatial memory by reducing the percent of alteration by 20% compared with AlCl_3_-intoxicated rats. On the other hand, treatment with *L. rhamnosus* showed enhancement in both learning and memory tasks by reducing the escaping latency by 26% and increasing the time spent in the target quadrant by about 3.2 times compared with AlCl_3_-intoxicated rats. In addition, treatment with *L. rhamnosus* reduced the percent of AlCl_3_-alteration by 21% compared with AlCl_3_-intoxicated rats. Co-administration of both sesamol and *L. rhamnosus* reduced the alteration in learning and memory tasks by 29% and 96%, respectively, compared with the AlCl_3_-intoxicated rats (i.e., the learning task became more or less non-significantly different than the control group). In addition, co-administration of both sesamol and *L. rhamnosus* reduced the alteration in spatial memory by 96% compared with AlCl_3_-intoxicated rats (i.e., the learning task became more or less non-significantly different than the control group). Moreover, this indicates that co-administration of both sesamol and *L. rhamnosus* has an augmenting effect against the AlCl_3_ deleterious effect against memory tasks and spatial memory, leading to the restoration of their normal function without any significant difference from normal.

### 3.3 Effect of administration of sesamol and/or *L. rhamnosus* on the histopathological changes in different brain and liver tissues of AlCl_3_-intoxicated rats

**FIGURE 4:**
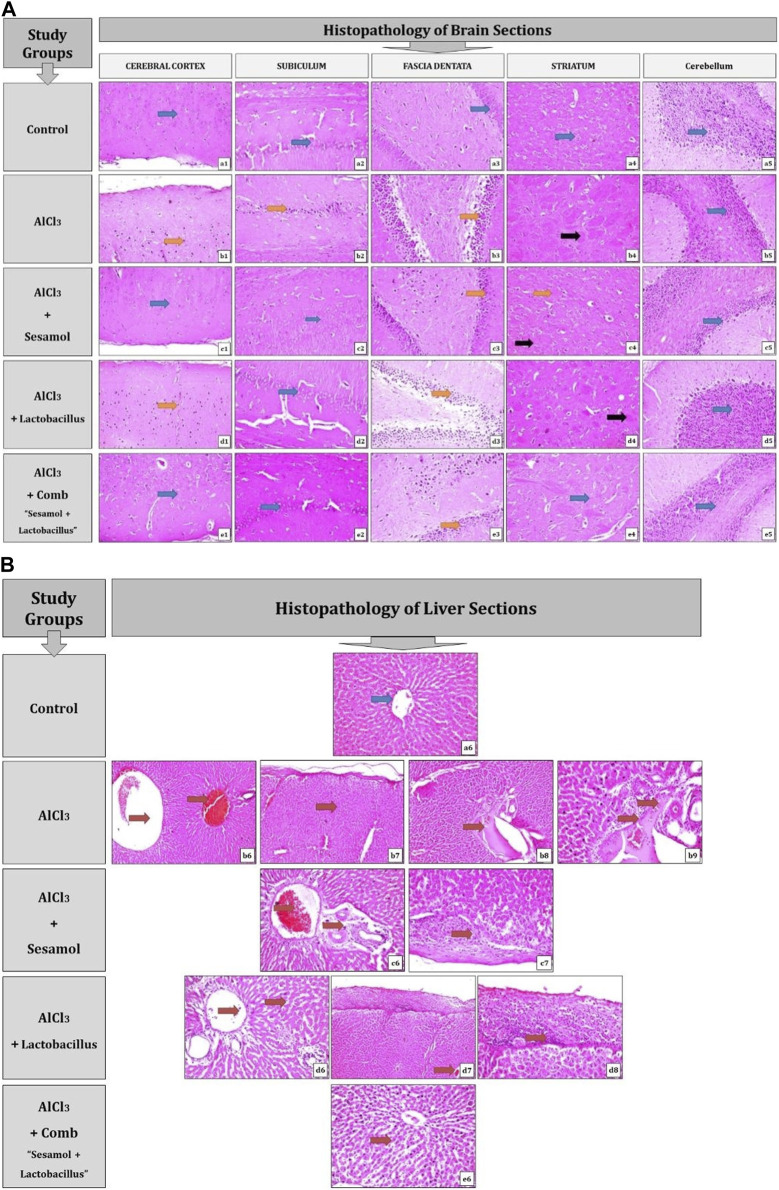
Photomicrographs showing regions of the brain (cerebral cortex, subiculum, and fascia dentata in the hippocampus, striatum, and cerebellum areas) **(A)** and liver sections **(B)** stained by the hematoxylin and eosin strain (magnification ×40). a1, a2, a3, a4, a5, and a6: control group; b1, b2, b3, b4, b5, b6, b7, b8, and b9: AlCl_3_ group; c1, c2, c3, c4, c5, c6, and c7: AlCl_3_ + sesamol group; d1, d2, d3, d4, d5, d6, d7, and d8: AlCl_3_ + *Lactobacillus rhamnosus* group; and e1, e2, e3 e4, e5, and e6: AlCl_3_ + combination group.

As shown in Figure 4A and Table 2, the results for brain sections show that, in the control group, there was no histopathological alteration, and the normal neuronal structure was observed in all regions of the brain (cerebral cortex, subiculum, and fascia dentata in hippocampus, striatum, and cerebellum areas) (insets a1, a2, a3, a4, and a5). In the AlCl_3_ group, nuclear pyknosis and degeneration were detected in all neurons of the cerebral cortex area (inset b1), in the fascia dentata (inset b2), and in ]subiculum areas ( inset b3) of the hippocampus. In addition, multiple large-sized focal eosinophilic plaque formations with loss of neurons were detected in the striatum area of the brain (inset b4). However, there were no histopathological changes recorded in the cerebellum area (inset b5). In the AlCl_3_ + sesamol group, there was no histopathological alteration recorded in the cerebral cortex (inset c1) or subiculum of the hippocampus (inset c2). Some neurons displayed nuclear pyknosis and degeneration in the fascia dentata of the hippocampus (inset c3). Moreover, several striatal neurons formed a few localized, small-sized eosinophilic plaques with nuclear pyknosis. (inset c4). There were no histopathological changes observed in the cerebellum (inset c5). In the AlCl_3_ + *L. rhamnosus* group, all the neurons in the cerebral cortex had nuclear pyknosis and degeneration (inset d1) and in the fascia dentata area of the hippocampus (inset d3). However, there were no histological changes found in the subiculum (inset d2) or cerebellum (inset d5). Additionally, focal small size eosinophilic plaque formation with nuclear pyknosis in most of the neurons was recorded in the striatum area of the brain (inset d4). In the AlCl_3_ + sesamol + *L. rhamnosus* group, there was no histopathological alteration as recorded in the cerebral cortex (inset e1), subiculum of the hippocampus (inset e2), striatum (inset e4), and cerebellum (inset e5). Yet, most of the neurons in the fascia dentata of the hippocampus had nuclear pyknosis and degeneration (inset e3).

**TABLE 2:** Severity of histopathological alterations in brain areas of the different experimental groups.

	Control	AlCl_3_	AlCl_3_ + sesamol	AlCl_3_ + *L. rhamnosus*	AlCl_3_ + combination
Degeneration and nuclear pyknosis in the neuronal cells of cerebral cortex	−	+++	−	+	−
Degeneration and nuclear pyknosis in the hippocampal subiculum	−	+++	−	−	−
Degeneration and nuclear pyknosis in the hippocampal neuronal cell fascia	−	+++	++	+	+
Focal eosinophilic plaques were detected in the striatum	−	+++	+	+	−

− = zero normal histological status without changes, + = minimal changes less than 25% of examined tissue sections including changes, ++ = moderate changes about 25–50% and +++ = severe changes more than 50% of examined tissue sections have changes.

As illustrated in Figure 4B and Table 3, the findings from liver sections demonstrate that there was no histopathological change in the control group, and typical histology of the central vein and the surrounding hepatocytes was noted (inset a6). In the AlCl_3_ group, dilatation and congestion were observed in the central and portal veins (inset b6), associated with thickening in the Glisson’s capsule (inset b7). In the portal region, there were numerous freshly developed bile ductuli and dilated portal veins (inset b8). Infiltration of inflammatory cells was visible in the portal area (inset b9). In the AlCl_3_ + sesamol group, there was congestion in the portal vein associated with edema in the portal area (inset c6). Glisson’s capsule showed thickening in the histological structure by inflammatory cells (inset c7). In the AlCl_3_ + *L. rhamnosus* group, dilatation and congestion were detected in portal vein and sinusoids associated with focal inflammatory cells in the parenchyma (inset d6). There was thickening in the Glisson’s capsule (inset d7, d8). In the AlCl_3_ + sesamol + *L. rhamnosus* group, there was only dilatation and congestion in the central veins and sinusoids (inset e6).

**TABLE 3:** Severity of histopathological alterations in liver areas of different experimental groups.

	Control	AlCl_3_	AlCl_3_ + sesamol	AlCl_3_ + *Lactobacillus rhamnosus*	AlCl_3_ + combination
Central and portal vein dilatations and congestion	−	+++	+	+	+
Thickening in the Glisson’s capsule	−	+++	+	+	−
Several newly formed bile ductulus in the portal area with dilatation in the portal vein	−	+++	−	−	−
Inflammatory cell infiltration in the portal area	−	+++	−	−	−

− = zero normal histological status without changes, + = minimal changes less than 25% of examined tissue sections including changes, ++ = moderate changes about 25–50% and +++ = severe changes more than 50% of examined tissue sections have changes.

In brain sections, nuclear pyknosis and degeneration are indicated by the orange arrow, there are no histological changes indicated by the blue arrow, and localized eosinophilic plaques are indicated by the black arrow.

In liver sections, the blue arrow denotes the absence of any histopathological changes, and the red arrow denotes any documented histological changes, such as the dilatation and congestion of the central and portal veins or the infiltration of inflammatory cells.

### 3.4 Effect of administration of sesamol and/or *L. rhamnosus* on the brain contents of β-catenin, Wnt3a, and GSK-3β in AlCl_3_-intoxicated rats

**FIGURE 5:**
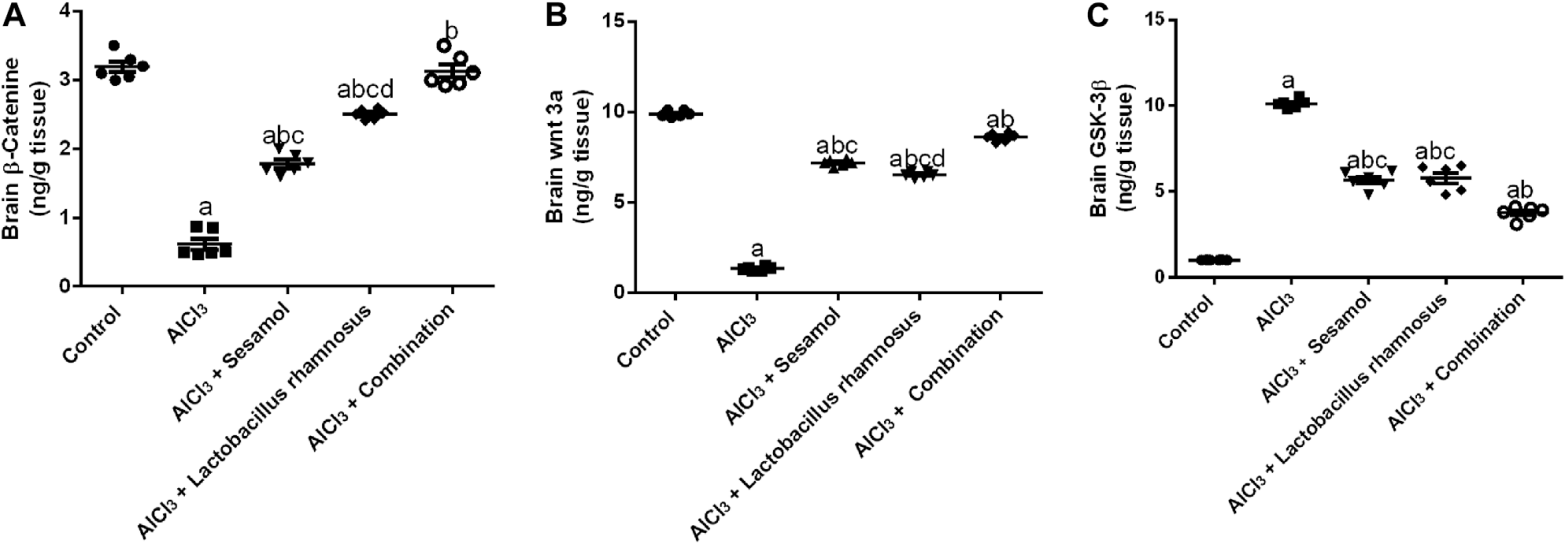
Effect of administration of sesamol and/or *Lactobacillus rhamnosus* on the brain contents of β-catenin **(A)**, Wnt3a **(B)**, and GSK-3β **(C)** in AlCl_3_-intoxicated rats. Values are showed as the mean (*n* = 6) ± SEM. One-way ANOVA was used for the statistical analysis, followed by Tukey’s *post hoc* test. Comparison of (a) control, (b) AlCl_3_, (c) sesamol + *Lactobacillus rhamnosus*, and (d) *sesamol* treated groups; *p* < 0.05.

AlCl_3_-intoxicated rats showed a significantly reduced level of free β-catenin (Figure 5A), about 82%, and the level of Wnt3a (Figure 5B), about 78%. Meanwhile, AlCl_3_-intoxicated rats showed a significantly elevated level of GSK-3β (Figure 5C), about 10 times higher compared to control rats. Treatment with sesamol only elevated the level of β-catenin by 2.3 times and the level of Wnt3a by 7.6 times compared with AlCl_3_-intoxicated rats. Meanwhile, it reduced the elevated level of GSK-3β by 25% compared with AlCl_3_-intoxicated rats. In the same way, treatment with *L. rhamnosus* significantly elevated the level of β-catenin by 3.5 times and the level of Wnt3a by 7.4 times compared with AlCl_3_-intoxicated rats. Meanwhile, it reduced the levels of GSK-3β by 28% compared with AlCl_3_-intoxicated rats. Co-administration of both sesamol and *L. rhamnosus* has an augmenting effect against the AlCl_3_-induced decline in levels of both free β-catenin and Wnt3a, restoring them to their normal levels, and against the elevated level of GSK-3β as compared to AlCl_3_-intoxicated rats.

### 3.5 Effect of administration of sesamol and/or the probiotic bacteria *L. rhamnosus* on the expressions of inflammatory, TNF-α, NF-κB, IL-1β, IL-6β, and apoptotic, Bax, Bcl2, and caspase-3 biomarkers in AlCl_3_-intoxicated rats in both brain and liver tissues

**FIGURE 6:**
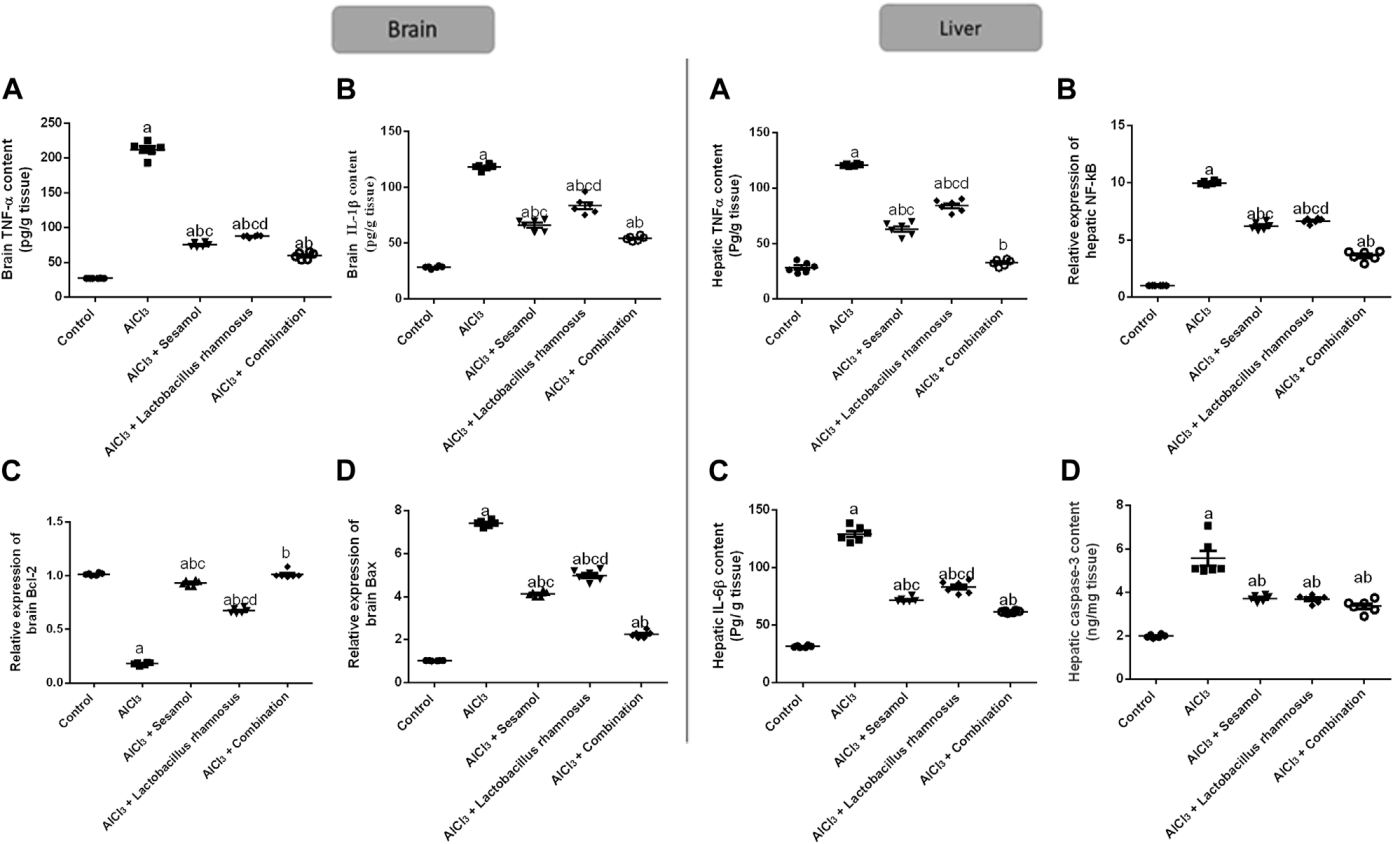
Effect of administration of sesamol and/or *Lactobacillus rhamnosus* on the expressions of inflammatory, TNF-α, NF-_k_β, IL-1β, and IL-6β, and apoptotic, Bax and Bcl2, and caspase-3 biomarkers in AlCl_3_-intoxicated rats in both the brain (panel I) [**(A)** TNF-α, **(B)** IL-1β, **(C)** Bcl2, and **(D)** Bax] and liver (panel II) [**(A)** TNF-α, **(B)** IL-6β, **(C)** NF-κB, and **(D)** caspase-3] tissues. Values are presented as the mean (*n* = 6) ± SEM. One-way ANOVA was used for the statistical analysis, followed by Tukey’s *post hoc* test. Comparison of (a) control, (b) AlCl_3_, (c) sesamol + *Lactobacillus rhamnosus*, and (d) *sesamol* treated groups; *p* < 0.05. IL-6, interleukin-6; NF-κB, nuclear-factor kappa b; TNF-α, tumor necrosis factor-alpha.

As demonstrated in Figure 6, AlCl_3_-intoxicated rats upregulated the brain inflammatory response, TNF-α and IL-1β, about 7.4 and 2.9 times, respectively (panel I; Figure 6A, B), which was extended peripherally to the liver; TNF-α and IL-6β, about 2.87 and 3.1 times, respectively (panel II; Figure 6A, B), as compared to the control group. This inflammatory response was linked to the increase in central and peripheral, and in the liver, programmed cell death, as affirmed by the significant downregulation of the anti-apoptotic marker *Bcl2* mRNA by 79% and the significant upregulation of the apoptotic marker Bax mRNA in brain tissues by 7.8 times compared with the control group (panel I; Figure 6C, D). Moreover, hepatic TNF-α and caspase-3 levels were significantly enhanced by 10 and 2.89 times, respectively, compared with the control group (panel II; Figures 6C, D). Treatment with sesamol reduced the elevated levels of TNF-α and IL-1β by 67% and 49%, respectively, in brain tissues compared with AlCl_3_-intoxicated rats. Contrariwise, treatment with *L. rhamnosus* significantly reduced the levels of TNF-α and IL-1β by 47% and 34%, respectively, in brain tissues compared with AlCl_3_-intoxicated rats. Co-administration of both sesamol and *L. rhamnosus* has an augmenting effect against the AlCl_3_-induced elevated levels of TNF-α and IL-1β in brain tissues by 71% and 50%, respectively, as compared to the AlCl_3_-intoxicated group. Furthermore, treatment with sesamol only increased the reduced level of brain Bcl2 mRNA, restoring it to normal values. Meanwhile, treatment with *L. rhamnosus* reduced the elevated level of Bax mRNA by 21%. However, co-administration of both sesamol + *L. rhamnosus* restored the level of brain *Bcl2* mRNA to normal values and decreased the level of Bax mRNA by 80%.

The deleterious effect of AlCl_3_ on the liver was evaluated and compared to the control group. Treatment with sesamol only reduced the elevated levels of hepatic TNF-α, IL-6β, NF-κβ, and caspase-3 by 49, 62, 33%, and 29%, respectively, compared with AlCl_3_-intoxicated rats. In the same way, treatment with *L. rhamnosus* significantly reduced the levels of hepatic TNF-α, IL-6β, NF-_K_β, and caspase-3 by 26%, 36%, 31%, and 28% compared with AlCl_3_-intoxicated rats. Co-administration of both sesamol and *L. rhamnosus* has an augmenting effect against the AlCl_3_-induced elevated levels of hepatic TNF-α, IL-6β, NF-κB, and caspase-3 by 89%, 68%, 61%, and 31% in hepatic tissues compared with AlCl_3_-intoxicated rats. This indicates that co-administration of both sesamol and *L. rhamnosus* has an augmenting effect against the AlCl_3_-induced inflammatory and apoptotic effects both centrally and peripherally.

### 3.6 Effect of administration of sesamol and/or *L. rhamnosus* on the hepatic mRNA expression of JAK-2 and STAT-3 in AlCl_3_-intoxicated rats

**FIGURE 7:**
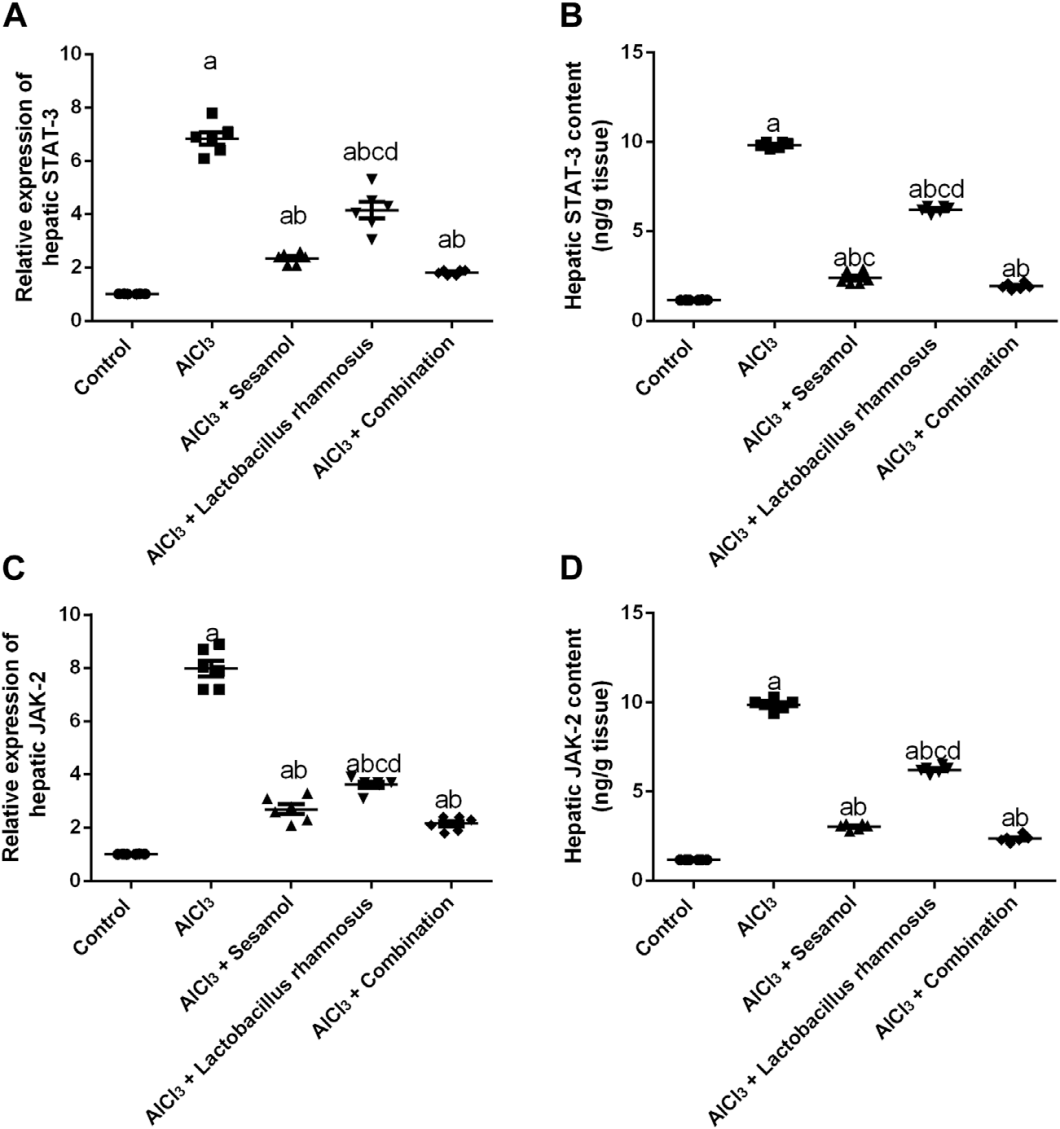
Effect of administration of sesamol and/or *Lactobacillus rhamnosus* on the hepatic mRNA expressions and hepatic levels of STAT-3 **(A,B)** and JAK-2 **(C,D)** in AlCl_3_-intoxicated rats. Values are presented as the mean (*n* = 6) ± SEM. Statistical analysis was carried out using the one-way ANOVA followed by Tukey’s *post hoc* test. Comparison of (a) control, (b) AlCl_3_, (c) sesamol + *Lactobacillus rhamnosus*, and (d) *Sesamol* treated groups; *p* < 0.05. JAK, Janus kinase; STAT: signal transducer and activator of transcription.

The hepatic mRNA expression of STAT-3 and hepatic STAT-3 levels (Figures 7A, B) were significantly increased by 6.8 and 8.4 folds, respectively, and the hepatic mRNA expression of JAK-2 and hepatic JAK-2 levels (Figures 7C, D) were significantly increased by 7.9 and 8.4 folds, respectively, in AlCl_3_-intoxicated rats compared to the control group. These results showed the deleterious effect of AlCl_3_ on the liver as compared to the control group. Furthermore, treatment with sesamol alone resulted in a significant downregulation of the mRNA expression and hepatic level of STAT-3 (67.3% and 75.6%, respectively), together with both the mRNA expression and hepatic level of JAK-2 (87% and 69%, respectively), compared with AlCl_3_-intoxicated group. Meanwhile, treatment with *L. rhamnosus* alone reduced the elevated mRNA expressions and hepatic levels of both STAT-3 (36.7% and 36.8%, respectively) and JAK-2 (48% and 37%, respectively) compared with the AlCl_3_-intoxicated group. Co-administration of both sesamol and *L. rhamnosus* reduced the elevated mRNA expressions and hepatic levels of both STAT-3 (74.3% and 80.3%, respectively) and JAK-2 (87.8% and 76%, respectively) compared with the AlCl_3_-intoxicated group.

### 3.7 Effect of administration of sesamol and/or *L. rhamnosus* on hepatic fibrotic biomarkers; (MMP-2, TIMP-1, and α-SMA) and hepatic PPAR-γ in AlCl_3_-intoxicated rats

**FIGURE 8:**
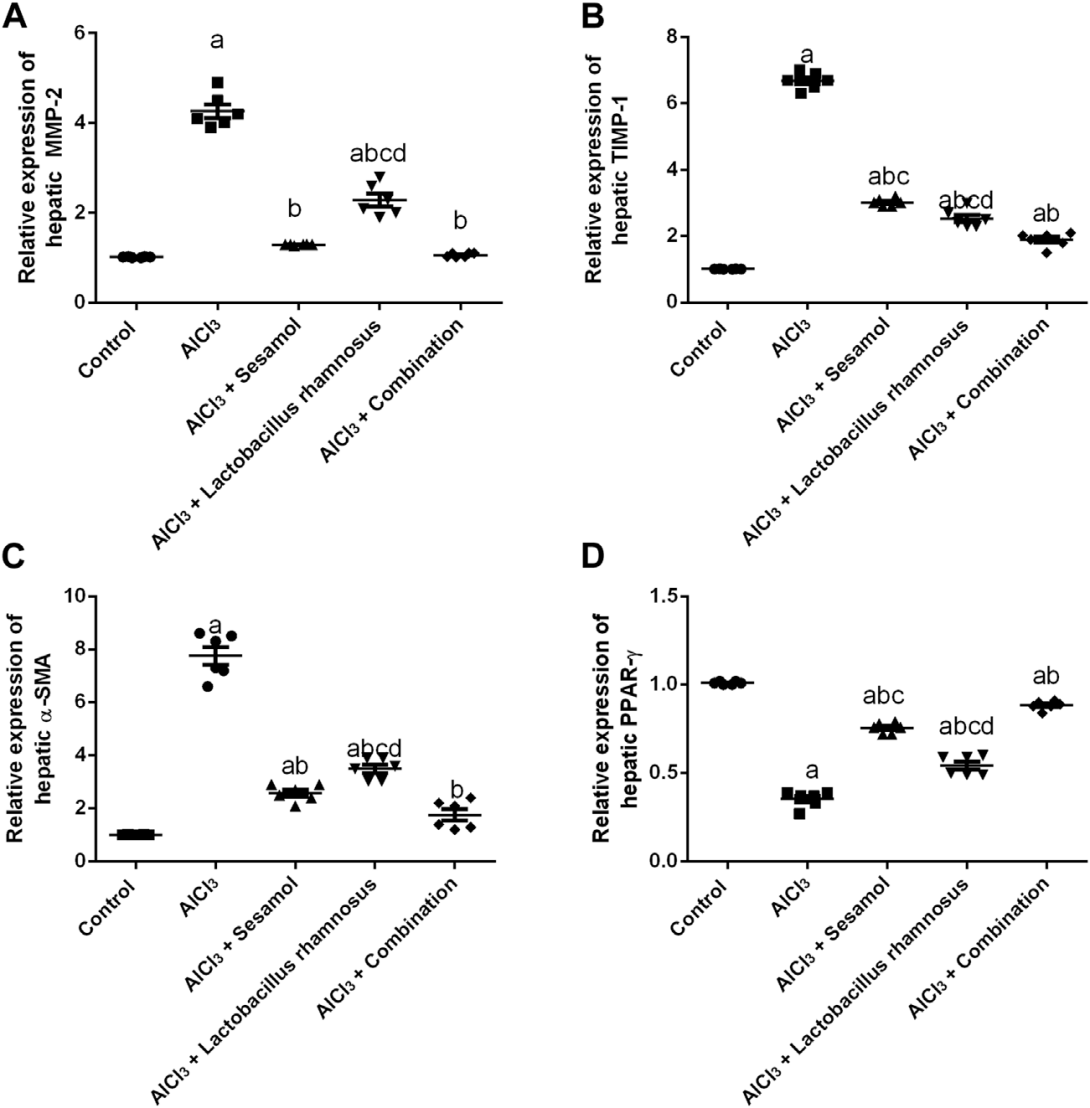
Effect of administration of sesamol and/or *Lactobacillus rhamnosus* on hepatic fibrotic biomarkers [MMP-2 **(A)**, TIMP-1 **(B),** and α-SMA **(C)**] and hepatic PPAR-γ **(D)** in AlCl_3_-intoxicated rats. Values are presented as the mean (*n* = 6) ± SEM. One-way ANOVA was used for the statistical analysis, followed by Tukey’s *post hoc* test. Comparison of (a) control, (b) AlCl_3_, (c) sesamol + *Lactobacillus rhamnosus*, and (d) *sesamol* treated groups; *p* < 0.05. α-SMA, alpha-smooth muscle actin; MMP-2, matrix metalloproteinase-2, PPAR-γ, peroxisome proliferator-activated receptor gamma; TIMP-1, tissue inhibitor matrix metalloproteinase-1.

Rats intoxicated with AlCl_3_ have significantly elevated levels of both the hepatic proteins MMP-2 (Figure 8A) and TIMP1 (Figure 8B) (4.3 and 5.4 times, respectively) compared with the control group. These results evaluated the deleterious effect of AlCl_3_ on the liver as compared to the control group. Treatment with sesamol alone significantly downregulated the increased hepatic levels of both MMP-2 and TIMP-1 by 66% and 57%, respectively, compared with AlCl_3_-intoxicated rats. In the same way, treatment with *L. rhamnosus* alone also significantly decreased the hepatic levels of both MMP-2 and TIMP-1 by 35% and 55%, respectively, compared with AlCl_3_-intoxicated rats. However, co-administration of both sesamol and *L. rhamnosus* restored the protein expression of MMP-2 to its normal levels and decreased the hepatic increased level of TIMP-1 by 69.1% as compared with AlCl_3_-intoxicated rats.

Meanwhile, the AlCl_3_-intoxicated group exhibited a significant decline in hepatic PPAR-γ protein expression (60%) (Figure 8C) and a significant increase in hepatic α-SMA protein expression (8 times) (Figure 8D). These results evaluated the deleterious effect of AlCl_3_ on the liver as compared to the control group. Meanwhile, treatment with sesamol alone significantly upregulated the level of PPAR-γ twice and significantly reduced the α-SMA level by 75% compared with the AlCl_3_-intoxicated group. Treatment with *L. rhamnosus* alone also significantly elevated the hepatic level of PPAR-γ by 1.3 times and significantly reduced the hepatic protein level of α-SMA by 50% compared with the AlCl_3_-intoxicated group. However, co-administration of both sesamol and *L. rhamnosus* restored the protein expression of both PPAR-γ and α-SMA to their normal levels as compared with AlCl_3_-intoxicated rats. Moreover, this indicates that co-administration of both sesamol and *L. rhamnosus* has an augmenting effect against peripheral fibrotic and inflammatory activities in liver tissues.

### 3.8 Effect of administration of sesamol and/or *L. rhamnosus* on the hepatic enzymes ALT, AST, and total bilirubin in AlCl_3_-intoxicated rats

**FIGURE 9:**
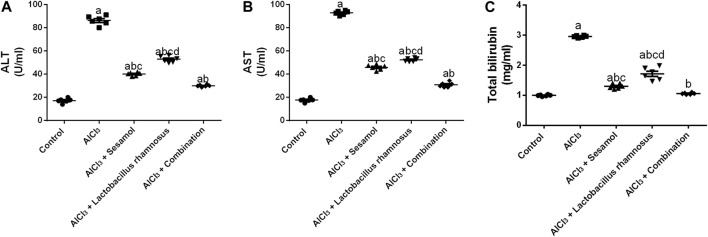
Effect of administration of sesamol and/or *Lactobacillus rhamnosus* on the hepatic enzymes ALT **(A)**, AST **(B)**, and total bilirubin **(C)** in AlCl_3_-intoxicated rats. Values are presented as the mean (*n* = 6) ± SEM. Statistical analysis was carried out using the one-way ANOVA followed by Tukey’s *post hoc* test. Comparison of (a) control, (b) AlCl_3_, (c) sesamol + *Lactobacillus rhamnosus*, and (d) *sesamol*-treated groups; *p* < 0.05. ALT, alanine aminotransferase; AST; aspartate aminotransferase.

Rats intoxicated with AlCl_3_ have significantly elevated levels of hepatic enzymes, ALT (Figure 9A), AST (Figure 9B), and total bilirubin (Figure 9C), by 4.68, 5.14, and 2 times, respectively, compared with the control group. These results evaluated the deleterious effect of AlCl_3_ on the liver as compared to the control group. Treatment with sesamol alone significantly downregulated the hepatic liver enzyme levels of ALT, AST, and total bilirubin by 56.1%, 51%, and 53.3%, respectively, compared with AlCl_3_-intoxicated rats. Treatment with *L. rhamnosus* alone also significantly decreased the hepatic liver enzyme levels of ALT, AST, and total bilirubin by 41.6%, 42.7%, and 29.8%, respectively, compared with AlCl_3_-intoxicated rats. However, co-administration of both sesamol and *L. rhamnosus* restored the hepatic level of total bilirubin to its normal levels and decreased the hepatic enzyme levels of both ALT and AST by 65.2% and 68.8%, respectively, as compared with AlCl_3_-intoxicated rats. This indicates that co-administration of both sesamol and *L. rhamnosus* has an augmenting effect on liver protection.

## 4 Discussion

Despite the hazardous consequences of long-term exposure, Al cookware is still being used in rural areas owing to its low price. Al is highly distributed in the liver and brain tissues, leading to neurotoxicity, neurodegenerative diseases, and liver diseases (Congdon and Sigurdsson, 2018; Zhu et al., 2020). Using probiotics as an add-on therapy with herbal products may be regarded a promising avenue for attenuation of Al-induced neurotoxicity and neurodegenerative diseases. Herein, we investigated the protective effects of sesamol, probiotics, and their combination against Al-induced deleterious effects on the brain and liver, highlighting the mechanistic pathways contributing to their ameliorative effects.

Al neurotoxicity contributes to the accumulation of amyloid-β (Aβ) and the hyperphosphorylated tau (p-tau) protein (Hussien et al., 2018)**.** Consequently, we measured Aβ and the p-tau protein as markers for evaluating the neuroprotective ability of the chosen drugs in order to develop a drug or regimen that can reduce the formation of Aβ or improve its clearance. Treatment with sesamol and/or the probiotic *Lactobacillus rhamnosus (L. rhamnosus*) showed anti-dementia properties by reducing the elevated dementia markers, and their combination showed the best results among the treatment regimens in comparison with the AlCl_3_-intoxicated group. These results are in agreement with previous studies (Ohsawa et al., 2015; Liu et al., 2017b).

In the current study, Al-intoxicated rats revealed impairment in learning and memory, as well as lower activity and exploration, as depicted by MWM and Y-maze tests, respectively. These findings are consistent with publications by John et al. (2015), Thenmozhi et al. (2015), Soubh et al. (2021). Such behavioral disturbance was confirmed by the alteration of inflammatory, pro-inflammatory, and apoptotic biomarkers. The abnormal assemblies of Aβ and excessive phosphorylation of Tau were proven to affect neuronal plasticity and function, which are responsible for long-term behavioral disturbances (Soubh et al., 2021). Our proposed treatment with sesamol and/or *L. rhamnosus* (i.e., either monotherapy or combination therapy) prevented such deleterious behavioral effects of AlCl_3_. Sesamol showed the ability to reduce cognitive impairment caused by environmental pollutants (John et al., 2015) or stress exposure (Kumar et al., 2013), which is consistent with our findings. In addition, previous reports highlighted the potential of certain *Lactobacillus* strain supplementation as a probiotic in the regulation of emotional behavior in stress-related disorders (Bravo et al., 2011; Karen et al., 2021).

Downregulation of the Wnt signaling balance has been interconnected with the development of Al-induced neurotoxicity (Mohamed et al., 2022). Therefore, upregulation of the Wnt signaling pathway prevents the cytotoxic effects of the accumulated Aβ (Toledo and Inestrosa, 2010). In the current work, AlCl_3_ switched off the Wnt pathway as indicated by the upregulation of the level of GSK-3β, leading to the downregulation of free β-catenin. Sesamol alone or in combination with *L. rhamnosus* restored the basal levels of GSK-3β and β-catenin, halting the effects of AlCl_3_. This effect contributed to the behavioral enhancement and reversed the impaired synaptic plasticity and memory deficit, which goes along with the previously reported importance of activation of Wnt signaling for reducing neurological disorders in AD (Vargas et al., 2014)**.** To the best of our knowledge, there are no complete biochemical, histological, and behavioral studies for the effect of sesamol on the Wnt pathway in the AlCl_3_-intoxicated rat model. Nonetheless, downregulation of GSK-3β levels and modulation of the Wnt pathway are reported to be protective in many neurodegenerative diseases, including AD (Toledo and Inestrosa, 2010), which adds value to our study by using sesamol.

Additionally, switching off Wnt/β-catenin signaling plays a crucial role in the propagation of neuroinflammatory mediators. It was reported earlier that either Aβ aggregation or increased levels of GSK-3β activate the glial cells, with the subsequent production of inflammatory mediators, such as IL-6 and TNF-α (Majdalawieh and Ro, 2015; Gan et al., 2021). In our study, we found a significant increase in the level of inflammatory mediators both centrally in the brain and peripherally in the liver in rats after AlCl_3_ administration compared to the control group. However, sesamol alone or in combination with the chosen probiotic significantly decreased the unfavorable inflammatory condition in comparison to the AlCl_3_-intoxicated group. Consistent with our results, sesamol inhibited amyloidogenesis and inflammation-induced memory impairment by inhibiting NF-κB and the production of inflammatory mediators (Liu et al., 2017a). Furthermore, a former study that used a probiotic mixture containing our chosen strain, *L. rhamnosus*, presented a reduction in inflammatory markers, which evoked improvement in memory function in the AD model of rats (Ohsawa et al., 2015). It has been proven that probiotics have a strong ability to restore the homeostasis of the gut microbiota and ameliorate cognitive impairment through both anti-inflammatory and antioxidant pathways (Gibbs et al., 1999; Consolo et al., 2009; Couto et al., 2014). Our study proved that the use of probiotics showed a superior effect over sesamol as an anti-inflammatory agent both centrally and peripherally.

Neuronal loss in Al-induced neurotoxicity is closely linked with apoptosis, which could induce memory loss and cognitive impairment (Maya et al., 2016)**.** Notably, the accumulation of Aβ and p-tau proteins triggers apoptosis (Maurer et al., 2006; Green and Llambi, 2015; Wang et al., 2018). The accumulation of ROS may also initiate apoptosis (Hsin et al., 2008; Liu et al., 2020; Mesole et al., 2020). AlCl_3_ resulted in high levels of apoptotic markers, caspase-3 and Bax, and lower levels of the anti-apoptotic protein Bcl-2 in the brain and liver compared to normal rats. Our histological findings showed that the cerebral neurons of the Al-intoxicated group had undergone extensive neuronal death.

This study showed the ability of sesamol and or *L. rhamnosus* to prevent programmed cell death in brain and liver tissues, as marked herein by the reduced levels of the apoptotic markers *caspase-3* and *Bax*, together with an elevation in the anti-apoptotic marker *Bcl-2*, compared to the Al-intoxicated group. The anti-apoptotic power of sesamol has been previously reported (Hou et al., 2006; Gao et al., 2017). Sesamol’s anti-apoptotic effect in nerve cells may be mediated by molecular pathways that upregulate the anti-apoptotic protein Bcl-2 and inhibit the pro-apoptotic protein Bax (Abou-Zeid et al., 2021; Ruankham et al., 2021). As a result of sesamol’s antioxidant, anti-inflammatory, and anti-apoptotic properties, the administration of sesamol dramatically reduced Al-induced toxicity in the liver and brain. Additionally, *Lactobacillus helveticus* reduced lipopolysaccharide-induced hippocampus apoptosis via modulation of the gut–brain axis, implying that this probiotic and related strains may be useful in some neurodegenerative disorders (Mohammadi et al., 2019). Furthermore, *L. rhamnosus* has been shown to inhibit ROS release, enhance catalase, SOD, and BDNF levels, and regulate the *Bax*/*Bcl-2* ratio (Tian et al., 2020; Kumar et al., 2022). Our results go along with these data and give the histopathological and behavioral output of these molecular mechanisms. Dubey et al. (2019) showed that some species of *Lactobacillus* can chelate heavy metals in the intestine of mice, such as cobalt and lead. This may reduce the toxicity of these heavy metals and protect the intestinal microflora. In our model, we administered AlCl_3_ through the intraperitoneal route of administration. So, the main protection site is at the tissue level through the Wnt and inflammatory pathways. There is evidence of the ability of *L. rhamnosus* to chelate Al, a low-molecular-weight heavy metal. It may be an interesting point of research in the near future to study the effect of oral administration of *L. rhamnosus* on the Al serum and tissue levels.

In view of the notion that the liver is one of the most susceptible organs to the damaging effect induced by AlCl_3_, and owing to its essential role in the clearance of circulating Aβ, we chose to investigate the detrimental effects of Al on the liver, with highlights on the effect of this hepatic injury on neurotoxicity induced by AlCl_3_. Since the liver is a crucial participant in peripheral Aβ clearance, any disruption in this clearance could tip the delicate Aβ balance toward brain buildup. Moreover, increasing amyloid aggregation, neuroinflammation, apoptosis, and the disturbance in signaling molecules in different brain regions is thought to be connected to signals originating in the liver and gut microbiota; this is known as the gut microbiota–liver–brain axis (Daily et al., 2021). Previous reports showed that elevation in the AST/ALT ratio, a marker of liver function (Giannini et al., 1999), is associated with an elevation in circulating Aβ-40 levels (Wang et al., 2017), results that are quite related to ours.

One of the major pathways for inflammatory cytokine signal transduction is JAK-2/STAT-3 signaling (Nazeam et al., 2021). This cue affects cell proliferation, survival, development, and differentiation (Soubh et al., 2021). The AlCl_3_-intoxicated group showed significant stimulation of JAK-2 and its sub-molecule, STAT-3, in our investigation, which was provoked by a large increase in the amount of IL-6β, an inflammatory cytokine, in liver tissue. Our results are in agreement with those of Li et al., 2018. Moreover, the concurrent treatment with sesamol or *L. rhamnosus* and their combination inhibited this gush and prevented its hepatotoxic effect compared to the AlCl_3_-intoxicated group. Formerly, sesame seeds hampered thyroid cell growth via inhibition of STAT-3 translocation. Moreover, probiotics have been shown to reduce *Helicobacter pylori* infection by inhibiting JAK-2 via their anti-inflammatory properties (Lee et al., 2010).

Nuclear receptors like PPARs control variable pathways such as inflammatory responses and metabolic detoxification (Li and Guo, 2011). Thus, abnormal levels of PPARs are reported in several liver diseases (Peyrou et al., 2012). In the current work, we investigated PPAR-γ as one of the most important functional isoforms in the PPAR family that controls various cellular functions (Li and Guo, 2011; Tyagi et al., 2011; Peyrou et al., 2012), and AlCl_3_-intoxicated rats exhibited a marked decline in PPAR-γ expression compared to healthy rats. Previously, sesamol and sesame oil treatments were associated with upregulation of liver PPAR-γ (Majdalawieh and Ro, 2015). This is consistent with our results, which proved that sesamol administration restored the contents of PPAR-γ. In addition, administration of the probiotic *L. rhamnosus* was reported to increase the expression of PPAR-γ in the liver. Based on our data and previous studies, *L. rhamnosus* and sesamol alone have significant potential to reduce liver damage by inducing PPAR-γ activity and thus inflating their anti-inflammatory abilities (Lecarpentier and Vallée, 2016).

α-SMA is one of the markers that monitors liver fibrosis in patients with non-alcoholic fatty liver disease (NAFLD) (Di Cerbo et al., 2016; Patel et al., 2020). In this investigation, the administration of sesamol or *L. rhamnosus* alone or in combination resulted in considerable downregulation of α-SMA protein expression in the AlCl_3_-intoxicated group. According to the best of our knowledge, this is the first report on the effect of co-administration of sesamol and *L. rhamnosus* on α-SMA. The various anti-inflammatory potentials of both sesamol and *L. rhamnosus* were marked by inhibiting NF-κB, JAK-2/STAT-3 and increasing PPAR-γ hubs, which contributed to α-SMA downregulation; as α-SMA increases with chronic inflammation of the liver, these findings are in line with those of Periasamy et al. (2014) and Elalfy et al. (2019).

MMP-2 is one of the major enzymes that belong to the MMP family, which is a family of endopeptidase-dysregulated enzymes that increase during inflammation. On the other hand, TIMPs control the expression and activity of MMPs (Gibbs et al., 1999; Consolo et al., 2009). MMPs are reported to be found at various stages of hepatic illnesses, ranging from inflammation, liver injury, cirrhosis, fibrosis, and hepatocarcinogenesis to recovery and hepatic regeneration (Geervliet and Bansal, 2020).

In our study, there was a massive increase in liver expression of MMP-2 and TIMP-1, reflecting the inflammatory effect of AlCl_3_ on the liver. Administration of sesamol alone or with *L. rhamnosus* significantly ceased increased expressions of both MMP-2 and TIMP-1. Previous reports documented the ability of sesamol to hamper elevated MMP expression in various tissues (Lu et al., 2011; Periasamy et al., 2014). These findings provided plausible justification for the amelioration of AlCl_3_-mediated perturbation in liver function tests (AST, ALT, and bilirubin) by sesamol and/or *L. rhamnosus* documented herein.

In comparison to the AlCl_3_-intoxicated group, the hepatoprotective and neuroprotective powers of sesamol and/or *L. rhamnosus* were investigated, and they were able to preserve and correct hepatic and brain tissue scores by preventing histological abnormalities. Additionally, this tissue-preserving potential of both sesamol and *L. rhamnosus* can be attributed to their anti-dementia, anti-inflammatory, and anti-apoptotic properties and the modulation of various signaling molecules in both the brain and liver (Di Cerbo et al., 2016; Khadira Sereen et al., 2017; Ezdini et al., 2020).

## 5 Conclusion

Sesamol alone or in combination with the probiotic *L. rhamnosus* prevented cognitive dysfunction and preserved brain tissue, offering neuroprotection by anti-dementia, anti-inflammatory, and anti-apoptotic properties, modulation of the gut–liver–brain axis, Wnt/β-catenin/GSK-3β, inflammatory, and apoptotic pathways. In addition, these regimens, either single or combined, blocked the peripheral deleterious effect of AlCl_3_ on the liver, as exemplified herein by liver probing, where the hepatic expression of inflammatory mediators, apoptotic markers, fibrotic biomarkers, and markers of JAK-2/STAT-3 and PPAR-γ pathways was modulated, and hepatic histopathology was improved. Accordingly, we highly recommend performing clinical trials using sesamol and/or *L. rhamnosus* as add-on agents in the treatment protocol of Al-induced neurodegenerative diseases.

## Data Availability

The original contributions presented in the study are included in the article/Supplementary Materials; further inquiries can be directed to the corresponding authors.
