# Supplementary figures and images for: The protective effects of sesamol and/or the probiotic, Lactobacillus rhamnosus, against aluminum chloride-induced neurotoxicity and hepatotoxicity in rats: Modulation of Wnt/β-catenin/GSK-3β, JAK-2/STAT-3, PPAR-γ, inflammatory, and apoptotic pathways
Source: Front Pharmacol. 2023 Aug 2;14:1208252. doi: 10.3389/fphar.2023.1208252 (PMC10436218; doi:10.3389/fphar.2023.1208252)

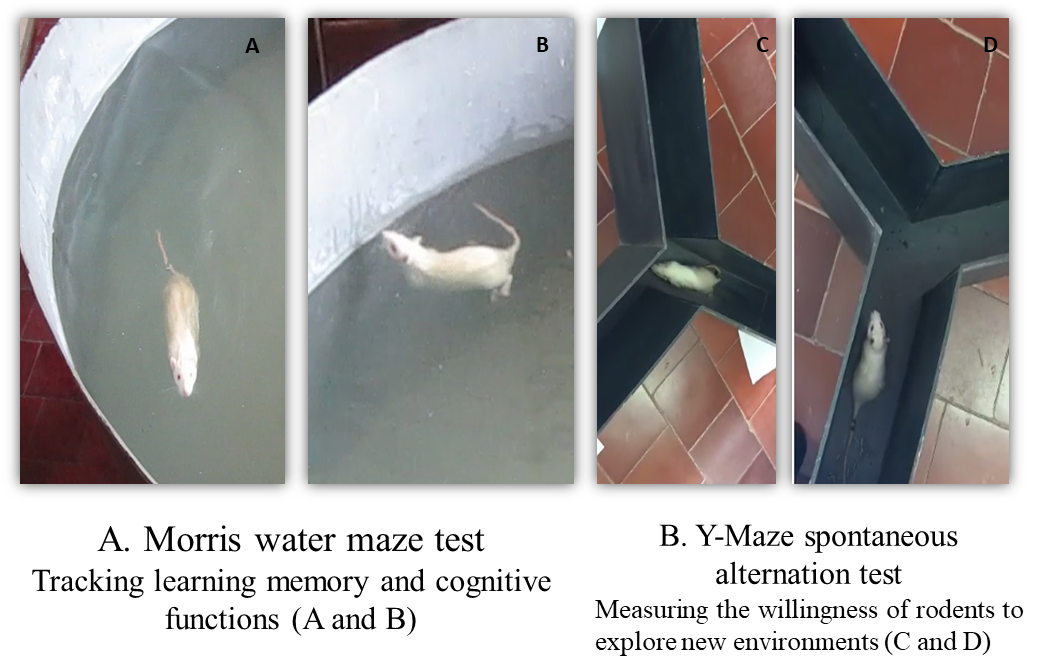

Supplement: Supplementary file 1 [file Image1.TIF]
